# A window-of-opportunity trial of the CXCR1/2 inhibitor reparixin in operable HER-2-negative breast cancer

**DOI:** 10.1186/s13058-019-1243-8

**Published:** 2020-01-10

**Authors:** Lori J. Goldstein, Raymond P. Perez, Denise Yardley, Linda K. Han, James M. Reuben, Hui Gao, Susan McCanna, Beth Butler, Pier Adelchi Ruffini, Yi Liu, Roberto R. Rosato, Jenny C. Chang

**Affiliations:** 1 Department of Medical Oncology, The Hospital of Fox Chase Cancer Center, Philadelphia, PA USA; 2 University of Kansas Medical Research Center, Fairway, KS USA; 3 Current address: Early Oncology Development, Bristol-Myers Squibb, 3401 Princeton Pike, Lawrenceville, NJ 08648 USA; 4 Tennessee Oncology, Nashville, TN USA; 5 Indiana University Simon Cancer Center, Indianapolis, IN USA; 6 Current address: Parkview Cancer Institute, 11141 Parkview Plaza, Suite 305A, Fort Wayne, IN 46845 USA; 7 Department of Hematopathology–Research, MD Anderson Cancer Center, Houston, TX USA; 8 Research and Development, Dompé farmaceutici S.p.A., 20122 Milan, Italy; 9 The Methodist Hospital Research Institute, 6445 Main Street, Houston, TX 77030 USA

**Keywords:** Cancer stem cells, CXCR1, Reparixin, Autophagy

## Abstract

**Background:**

Cancer stem cells (CSCs) are purported to be responsible for tumor initiation, treatment resistance, disease recurrence, and metastasis. CXCR1, one of the receptors for CXCL8, was identified on breast cancer (BC) CSCs. Reparixin, an investigational allosteric inhibitor of CXCR1, reduced the CSC content of human BC xenograft in mice.

**Methods:**

In this multicenter, single-arm trial, women with HER-2-negative operable BC received reparixin oral tablets 1000 mg three times daily for 21 days before surgery. Primary objectives evaluated the safety of reparixin and the effects of reparixin on CSC and tumor microenvironment in core biopsies taken at baseline and at treatment completion. Signal of activity was defined as a reduction of ≥ 20% in ALDH^+^ or CD24^−^/CD44^+^ CSC by flow cytometry, with consistent reduction by immunohistochemistry.

**Results:**

Twenty patients were enrolled and completed the study. There were no serious adverse reactions. CSC markers ALDH^+^ and CD24^−^/CD44^+^ measured by flow cytometry decreased by ≥ 20% in 4/17 and 9/17 evaluable patients, respectively. However, these results could not be confirmed by immunofluorescence due to the very low number of CSC.

**Conclusions:**

Reparixin appeared safe and well-tolerated. CSCs were reduced in several patients as measured by flow cytometry, suggesting targeting of CXCR1 on CSC.

**Clinical trial registration:**

Clinicaltrials.gov, NCT01861054. Registered on April 18, 2013.

## Background

Experimental models and retrospective clinical observations point to cancer stem cells (CSCs) as responsible for tumor initiation, treatment resistance, disease recurrence, and metastasis. Breast cancer was the first solid tumor where CSC was identified, and the markers used to identify them in this disease are CD24/CD44 [1] and aldehyde dehydrogenase (ALDH) [2]. CXCR1, one of the receptors for CXCL8 (IL-8), was identified on ALDH^+^ breast cancer CSC, and the addition of recombinant CXCL8 increased the CSC population and its propensity for invasion [3]. Binding of CXCL8 to CXCR1 on the CSC surface induces FAK phosphorylation. P-FAK, in turn, phosphorylates AKT and activates the Wnt pathway, which regulates stem cell renewal, and FOXO3A, which regulates cell survival. P-FAK also inhibits FADD, a downstream effector of FAS signaling, thereby sheltering CXCR1^+^ CSC from pro-apoptotic FASL-FAS interaction. Blocking of CXCR1 sensitizes CSC to FASL-mediated killing, thus making CXCR1 a targetable receptor on BC CSC [4]. Reparixin, an investigational allosteric inhibitor of CXCR1 and, to a lesser extent, of CXCR2 [5], reduced the metastatic spread of human BC cells and the CSC (both ALDH^+^ and CD24^−^/CD44^+^) content of human BC cell lines and xenografts in mice both as single agent and in combination with chemotherapy [4]. A CXCL8-CXCR1 axis in breast cancer CSC has been reported also by other investigators [6–8].

Tumor tissue is considered the gold standard for pharmacodynamics studies in solid tumors [9], and operable breast cancer represents an ideal setting to evaluate the functional and molecular effects of a novel agent in an unperturbed environment. In window-of-opportunity trials, patients with early-stage disease are treated for a brief “window” period with a novel agent followed by surgical resection [10]. Biopsies taken at the study entry and at the completion of treatment can be used to establish proof of concept. This trial design in principle suits the clinical testing of CSC-targeting agents allowing the enumeration as well as the isolation and functional characterization of CSC. These trials may also allow the potential to select for subsets of patients who might benefit from therapy in later-stage clinical trials, thus streamlining the clinical development of novel agents. However, the design of window-of-opportunity studies with non-cytotoxic agents in treatable patient populations may suffer from limitations such as small sample size and limited treatment duration to avoid any delay in surgery.

Reparixin was first tested in women with metastatic HER-2-negative BC in combination with weekly paclitaxel. A 30% response rate was recorded in 27 evaluable patients, with 2 long-lasting CR. The most frequent treatment-emergent adverse events (TEAEs) were gastrointestinal disorders (39% of all TEAEs), all grade ≤ 2. Grade 3 treatment-related TEAE was only 2.7% of all reports [11].

Thus, based upon preclinical [4] and clinical safety [11] data, a pilot, window-of-opportunity study was conducted to investigate the possibility that single-agent reparixin administration reduces CSC and induces modifications to the tumor microenvironment in breast cancer patients who were candidates for curative surgery.

## Methods and materials

### Patients

In this single-arm, monotherapy trial (NCT01861054), female patients aged ≥ 18 years with HER-2-negative operable breast cancer (with a clinical diameter of ≥ 2 cm) that were not candidates for neoadjuvant therapy were recruited. The protocol was amended later to allow inclusion of patients with HER-2-negative operable breast cancer with a clinical diameter of > 1 cm, not eligible for neoadjuvant treatment. Patients needed to have previously untreated, histologically proven (per local investigator assessment) ER+ and/or PgR+ or ER−/PgR− breast cancer (i.e., triple-negative BC (TNBC)) with adequate organ function.

### Study treatment

Patients were requested to take reparixin, an investigational orally available CXCR1/2 inhibitor, as two immediate-release 500-mg oral tablets every 6 to 8 h (every 8 h on the days of PK sampling) with food (a light meal or snack), for 21 consecutive days before surgery.

### Study objectives

The primary objective of this study was to evaluate the effects of orally administered reparixin on CSC in the primary tumor by measuring CSC by flow cytometry (FC) or RT-PCR and immunofluorescence (IF). Also, the study aimed at evaluating the effects of reparixin on the tumor microenvironment by measuring pathway markers, markers of angiogenesis and autophagy, and CXCR1 levels by IHC, and EpCAM and EMT markers by qRT-PCR. Last, systemic effects of reparixin were investigated by measuring the markers of inflammation in the plasma (Luminex-Multiplex) and polymorphonuclear neutrophil [PMN] biology in peripheral blood samples by FC. A co-primary objective of the study was to evaluate the safety of oral reparixin in the specific clinical setting. The secondary objective of this study was to define the pharmacokinetic (PK) profile of single-agent orally administered reparixin.

### Tumor biopsies

Patients underwent core biopsies at baseline (day − 14 to 0) and at the completion of therapy (day 21). Three to five ≥ 18-gage needle biopsies were taken to measure the changes in CSC populations and pathway markers. If not performed at the completion of treatment, a core biopsy was taken on the day of surgery by the same technique [12]. A sample of the surgically resected tumor tissue was also taken.

### Flow cytometry

CSC populations (CD24^−^/CD44^+^ and ALDH^+^) were measured by flow-cytometry of single-cell suspensions obtained from tumor biopsies (Additional file 1: Supplemental Materials) by using anti-human CD44-APC (clone G44-26) and CD24-FITC (clone ML5, RUO) antibodies (BD Biosciences, Franklin Lakes, NJ), and ALDEFLUOR assay (StemCell Technologies, Inc., Vancouver, BC, Canada), respectively, as previously published [13].

Additional analyses were directed to determine CXCR1 (PerCP/Cy5.5 mouse anti-human CD181 (CXCR1; BioLegend, San Diego, CA) and cell lineages using the following antibodies: APC mouse anti-human CD44 (BD Bioscience, San Jose, CA; catalog #559942), PE-Cy7 mouse anti-human CD24 (#561646), PE mouse anti-human CD2 (#555327), PE mouse anti-human CD31 (#555446), PE mouse anti-human CD3 (#555333), PE mouse anti-human CD18 (#555924), PE mouse anti-human CD16 (#555407), PE mouse anti-human CD19 (#555413), and PE mouse anti-human CD45 (#555483).

### Immunohistochemistry

All IHC analyses and scoring for extent (0 to 4) and intensity (0 to 3) were performed at the Houston Memorial Hermann Pathology laboratory as previously described [13]. After antigen retrieval (Tris-Cl, pH 9.0), paraffin-embedded sections of sample tumors were incubated for 1 h at room temperature with the following antibodies: anti-human CXCR1 (rabbit polyclonal to CXCR1 - C-terminal, ab137351; Abcam, Cambridge, MA), anti-human CD31 (clone JC70A, #IS610; Dako, Glostrup, Denmark), anti-human p62 (clone SQSTM1/p62, # 5114; Cell Signaling), anti-human AKT (rabbit polyclonal to pan-AKT, # ab8805; Abcam), anti-human FAK (clone pY861, #44-626G; Invitrogen, Qiagen, Carlsbad, CA), anti-human phospho-AKT (clone Ser473 (D9E), #4060; Cell Signaling), anti-human phospho-FAK (clone Y397, #ab4803; Abcam), and anti-human LC3B (clone D11, #3868; Cell Signaling). For the evaluation of extent, the following semi-quantitative score method (0 to 4) was used: 0, no positive cells; 1, 1–25%; 2, 26–50%; 3, 51–75%; and 4, 76–100%.

### RT-PCR

The analysis was performed by the Department of Hematopathology–Research, MD Anderson Cancer Center, Houston, TX, USA.

Core biopsy samples were placed in RNAlater, suspended in 700 μL of TRIzol® LS Reagent (Invitrogen) and homogenized using Bullet Blender (Next Advance Inc.). Next, RNA was extracted using the miRNeasy mini kit (Qiagen) and processed with the QIACube (Qiagen) automated low-throughput sample prep system. Thereafter, the isolated RNAs were reverse transcribed to cDNA (High Capacity cDNA Reverse Transcription Kit, ABI) and subsequently underwent quantitative RT-PCR (qRT-PCR) using 7900HT Fast Real-Time PCR System (ABI) to detect transcripts of markers associated with CSC (ALDH1, CD44, CD24, Akt, and PI3K), EMT (TWIST1, FOXC2, SNAIL1, SNAIL2, TG2, and ZEB1), epithelial cells (KRT19 and EpCAM), and leukocytes (CD45). We used average delta Ct (dCT) < 2 as a cutoff to determine the relative gene expression level. Since normal tissue was not available to act as the normalizer, we used GAPDH as the endogenous control (dCT = CT_target_ − CT_endogenous control_).

### Immunofluorescence

CSCs were evaluated also by IF at Houston Methodist Hospital (HMH) Pathology laboratory. Consecutive 3-μm sections were cut from each block for immunofluorescence experiments according to Liu et al. [14]. Incubation with primary antibodies against CD44 (mouse, #MS-668-R7, Thermo Scientific, USA), CD24 (mouse, #MS-1279-P1, Thermo Scientific), and ALDH1 (rabbit, #ab52492, Abcam) was followed by Alexa fluor 488-conjugated (green 500) anti-mouse IgG (H+L, #A11001, Invitrogen), Alexa fluor 647-conjugated (magenta-900) anti-mouse IgG (H+L; Jackson, USA), and Alexa fluor 546-conjugated (red-900) anti-rabbit IgG (H+L, #A11035, Invitrogen), respectively. DAPI staining (blue-200) was used to highlight the nucleus of the cells. Tumor cells with CD44 membrane staining (green) and ALDH1 (red) without membrane localization or co-localization of CD24 (magenta) were considered positive breast CSC. Immunofluorescence-positive controls included CD44+, ALDH1: human kidney tumor tissue: CD24+: human tonsil.

### Pharmacokinetics

Samples for pharmacokinetics (PK) were collected at selected sites in consenting patients. Venous blood samples (6 mL) were collected from a forearm vein at times 0.0, 0.25, 0.5, 1.0, 2.0, 4.0, 6.0, and 8.0 h following reparixin administration on days 1 and 21. The blood samples were immediately centrifuged at 4 °C, 1200 relative centrifugal force (RCF), for 10 min, and the plasma collected. Each plasma sample was divided into 2 aliquots and stored in 2 pre-labeled polypropylene screw-capped tubes (about 2 mL each) at − 20 °C until analysis. The analysis was performed at the Dompé Analytical Development Laboratories in L’Aquila, Italy.

### Statistics

In the absence of reference data from the literature, signal of activity was defined as a ≥ 20% reduction in CSC (defined by either the ALDH^+^− or CD24^−^/CD44^+^ phenotype) from baseline values as measured by flow cytometry, accompanied by a consistent reduction of the same cell population by IHC. The 20% cutoff was chosen considering only 1 21-day course of reparixin could be administered before surgery. Given the exploratory nature of the study to be conducted in a curable patient population, a sample of 40 patients divided into 2 subgroups of 20 patient each (i.e., ER+ and/or PgR+ and TNBC) was deemed adequate based upon simulations using the Wilson score method [15] to obtain a lower limit for the probability of success. For each subgroup with this sample size, the study will have 80% power to detect a signal of activity (as defined above) observed in ≥ 70% patients as statistically significant at the 0.05 significance level.

Descriptive statistics were used to summarize safety variables (i.e., adverse events, physical examination, vital signs, ECG, laboratory data, concomitant medications).

## Results

At the initiation of the study, escalating doses (i.e., 400 and 800 mg t.i.d.) of reparixin oral tablets had been tested in combination with weekly paclitaxel in the first 2 cohorts of a phase Ib clinical trial in metastatic HER-2-negative breast cancer [11] where a third cohort was to begin enrolling at 1200 mg t.i.d. dose level. One thousand milligrams t.i.d. represents an upper intermediate dose with respect to the phase Ib doses in combination. It was planned to enroll 2 subgroups of HER-2-negative patients into the study: group A, ER+ and/or PR+; group B, ER−/PR− (i.e., triple-negative breast cancer (TNBC)), and the sample size of 20 patients per group was deemed adequate. From May 2013 to November 2014, 20 patients in total were enrolled, 18 in croup A and 2 in group B, at 5 participating US sites. The study was closed prematurely due to slow enrollment in group B due to the widespread use of neoadjuvant chemotherapy in these patients. Patients’ main characteristics are reported in Table 1.

**Table 1:** Patient demographics

	HER-2 negative	
	ER+ and/or PgR+	ER− PgR−
*n*	18	2
Age, years, median (range)	52 (42–76)	56.5 (48–65)
Race, *n* (%)		
White	15 (83.3)	2 (100)
Black	1 (5.6)	0
Asian	1 (5.6)	0
Multiple	1 (5.6)	0
Weight, kg, median (range)	68.25 (55.3–97.2)	68.10 (56.7–79.5)
Clinical tumor stage		
IA	4 (22.2)	1 (50)
IB	3 (16.7)	0
T2N0M0 (IIa)	7 (38.9)	1 (50)
T2N1M0 (IIb)	2 (11.1)	0
T3N0M0 (IIb)	2 (11.1)	0

### Safety

All 20 patients completed the study treatment and were included in the safety population (i.e., all patients having taken at least 1 dose of the study treatment). No patients had to delay surgery due to study treatment. There were no treatment-emergent adverse events (TEAEs) leading to discontinuation. Fifteen of 20 patients experienced ≥ 1 TEAE. The most frequent TEAE was grade 1 fatigue (8 patients) and nausea (5 patients). Only 1 patient experienced a serious TEAE, unrelated to the study drug. Ten of 20 patients experienced ≥ 1 TEAE related to the study drug, all of which of grade ≤ 2. The most frequent TEAEs related to study drug were fatigue (4 patients) followed by nausea, headache, and flatulence (2 patients each).

It has been reported that CXCR2 inhibition may lead to transient, reversible neutropenia [16, 17]. Absolute neutrophil count (ANC) at weekly intervals was available for 10/20 patients, and neither neutropenia of any grade nor any sustained decrease in ANC was recorded in any patient at any time point (Additional file 1: Figure S1).

### Pharmacokinetics

**Fig. 1:**
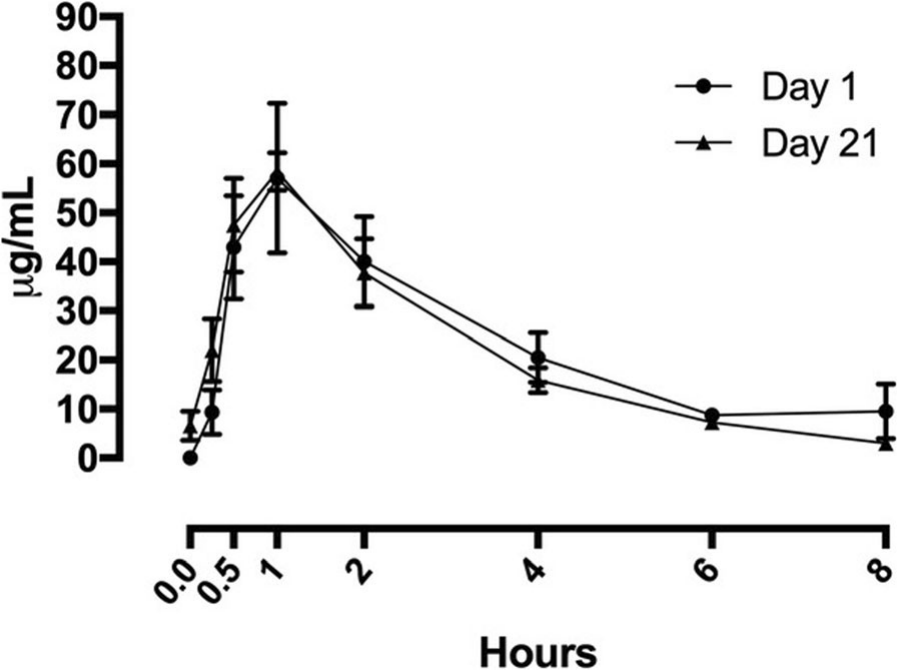
Mean total reparixin concentration versus time on day 1 (line with circles) and 21 (line with triangles). Results are presented as mean ± SEM

The PK population (i.e., consenting patients at select centers who received at least one dose of reparixin and having at least one valid, quantifiable PK parameter) consisted of six patients. Mean reparixin concentration versus time on day 1 and day 21 are shown in Fig. 1. Reparixin was rapidly absorbed after oral administration, with a median *t*_max_ of 1.0 h on both day 1 and day 21. Reparixin systemic exposure (*C*_max_ and AUC_last_) did not change from day 1 to day 21, indicating there is no accumulation upon multiple dosing. *t*_1/2_ did not vary from day 1 to day 21, with a median value of about 2 h.

### CSC

**Fig. 2:**
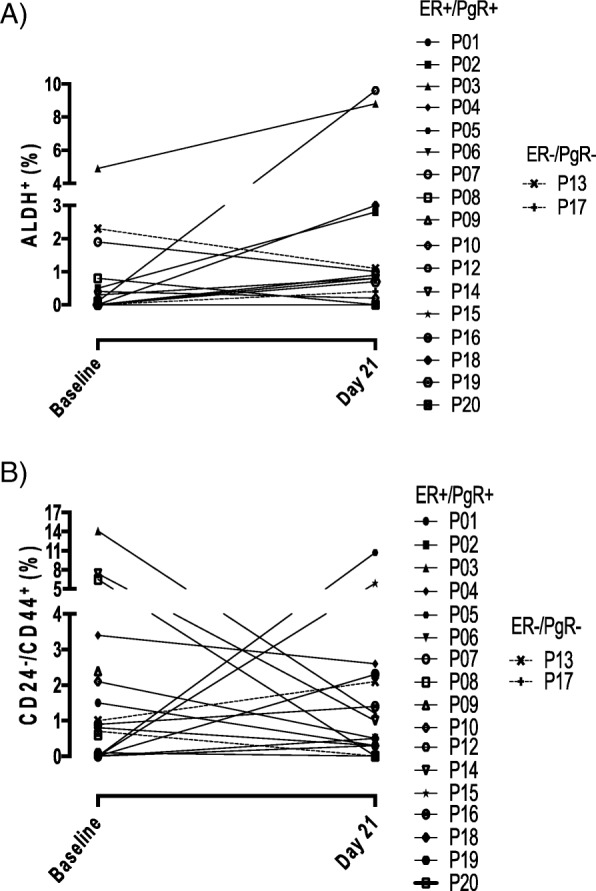
CSC evaluation in core biopsy samples by flow cytometry. **a** ALDH^+^ cells. **b** CD24^−^/CD44^+^ cells. Symbols represent individual patients. Patient 16’s day 21 core biopsy was taken on the day of surgery

The 2 largely non-overlapping populations of ALDH^+^ and CD24^−^/CD44^+^ CSC [2] were evaluated by flow cytometry using the gating strategy illustrated in Additional file 1: Figure S2. Since disparate information may be obtained from a core biopsy sample versus a surgery sample [12], Fig. 2 reports comparisons of core biopsy samples obtained before and after treatment. In 9/19 and 6/19 evaluable patients, there were neither ALDH^+^ nor CD24^−^/CD44^+^ CSC at baseline, respectively. In 3 patients, there were no CSCs of either phenotype in baseline samples. However, in 5/9 and 6/6 patients, CSCs of the ALDH^+^ or CD24^−^/CD44^+^ phenotype, respectively, were detected at day 21. A reduction of ≥ 20% in ALDH^+^ (Fig. 2a) or CD24^−^/CD44^+^ CSC (Fig. 2b) was recorded in 4 and 9 patients, respectively, out of 17 patients who had both baseline and day 21 core biopsy samples. In 6 patients, a decrease in CD24^−^/CD44^+^ CSC was not paralleled by a decrease in ALDH^+^ CSC, while in 2 patients, a decrease in ALDH^+^ cells was not accompanied by a reduction of CD24^−^/CD44^+^ cells. The very low number of CSC in tumor tissue hindered the possibility of confirming flow cytometry data by IF (Additional file 1: Figure S3). Fourteen patients provided tissues obtained by core biopsy at baseline and on day 21 of the study for the analysis of CSC markers (i.e., ALDH1, CD24, and CD44) by RT-PCR. Only 1/14 patients expressed ALDH1 transcripts at baseline, and all patients were negative for the same marker at day 21 (data not shown). Five patients had CSC (i.e., transcripts for CD44 but not for CD24) at both baseline and day 21 while 6 patients were negative for CSC at both baseline and day 21. Among the EMT-associated transcripts (Twist, FOXC2, SNAIL1, SNAIL2, TG2, and ZEB1), only TWIST was detected in 1 patient at baseline and in another patient on day 21. None of the other EMT-associated gene transcripts was detected in any patients at baseline or at day 21 of the study.

### CXCR1^+^ cells

**Fig. 3:**
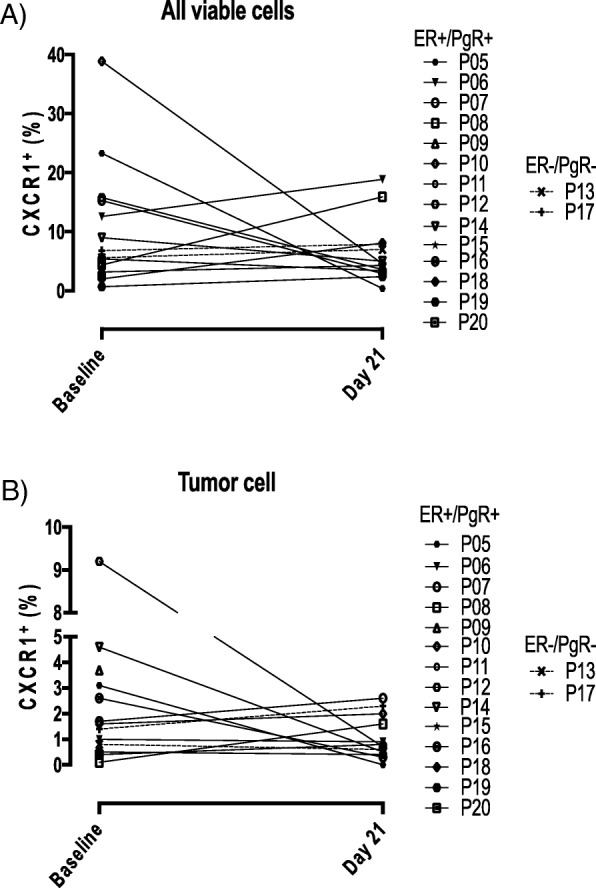
CXCR1^+^ cell evaluation in core biopsy samples by flow cytometry. **a** All viable cells. **b** Tumor cells. Symbols represent individual patients. Patient 16’s day 21 core biopsy was taken on the day of surgery

CXCR1/2 inhibition by reparixin has been shown in preclinical models to reduce the recruitment of CXCR1/2^+^ cells from the bloodstream to visceral sites [18]. Thus, we analyzed by flow cytometry both all viable CXCR1^+^ cells (i.e., PMN) and CXCR1^+^ tumor cells in core biopsy samples taken at baseline and day 21. All viable CXCR1^+^ cells were reduced at day 21 as compared with baseline in 6/13 evaluable patients (Fig. 3a). CXCR1^+^ tumor cells represented, as expected, a small percentage of cells (range 0.1–9.2%) in baseline samples [4] and were decreased at day 21 in 7/13 evaluable patients (Fig. 3b). In 4/13 evaluable patients, there was a parallel decrease in both CXCR1^+^ cell populations (Fig. 3a, b). A proportion of CD24^−^/CD44^+^ CSC stained positive for CXCR1 (JC Chang, personal communication), in keeping with the report by Ginestier and colleagues [4].

### Pathway markers

Considering the pleiotropic effects of CXCL8 in cancer (e.g., angiogenesis) [19, 20], and the hypothesized mechanism of action of CXCR1 inhibition on CSC [4], a number of markers were investigated on core biopsy samples by IHC.

At both baseline and day 21, the majority of patients had a value of 0 for the following markers: CD31 (16/20 baseline, 11/15 day 21), both extent and intensity; P-AKT (16/20 baseline, 11/15 day 21), both extent and intensity; LC3-B intensity; and P62 intensity.

**Fig. 4:**
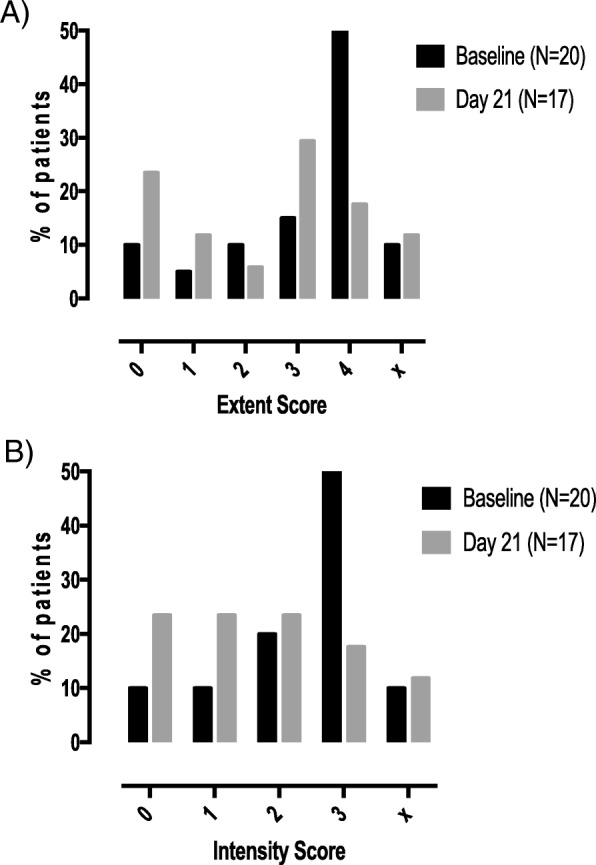
Change from baseline to day 21 in CXCR1 expression on tumor cells by IHC. The percentage of patients with each score is presented. **a** Extent. **b** Intensity. x, no tumor found on the block

A decrease in CXCR1 positivity, both in extent and intensity, was recorded at day 21 as the largest proportion of patients at baseline had results at levels 4 and 3, respectively, and level 3 and 2 or lower, respectively, at day 21 (Fig. 4).

**Fig. 5:**
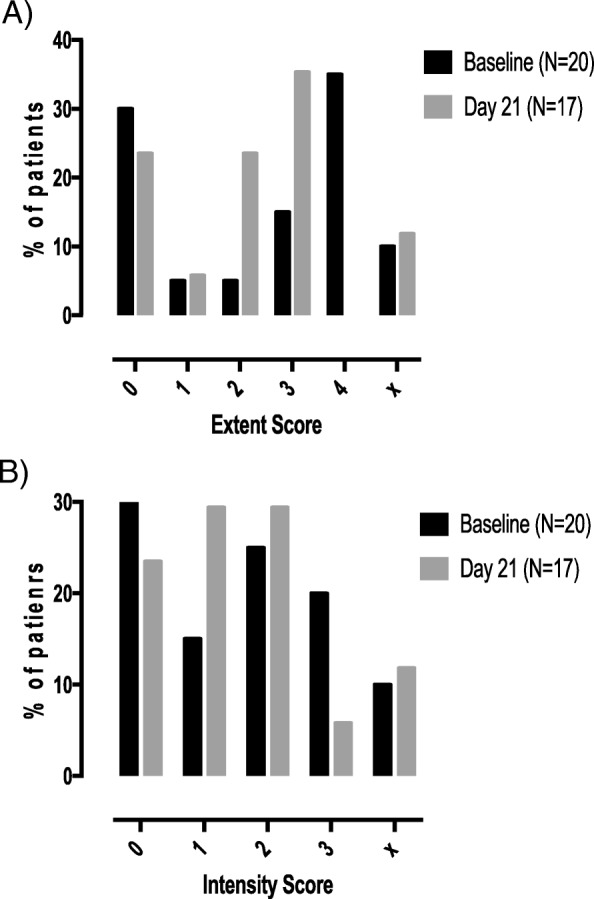
Change from baseline to day 21 in p62 expression on tumor cells by IHC. The percentage of patients with each score is presented. **a** Extent. **b** Intensity. x, no tumor found on the block

P62 extent (i.e., percentage of positive cells) decreased from level 4 to level 3 or lower in the majority of patients (Fig. 5), suggesting the induction of autophagy. The same pattern was observed for LC3B (data not shown).

### Cytokine levels

Considering the allosteric mechanism of CXCR1/2 inhibition, reparixin is not expected to block CXCL8 internalization following CXCL8-CXCR1/2 interaction; thus, reparixin administration is not expected to increase CXCL8 plasma levels. Individual patient CXCL8 serum levels (Additional file 1: Supplemental Materials) on days 1 and 21 are reported in Additional file 1: Figure S4. Baseline values are in line with those previously reported in healthy volunteers and early-stage cancer patients [21]. After 21 days of reparixin treatment, a small but significant (*p* < 0.05 by a non-parametric test, actual power only about 60% due to the small sample size) mean/median CXCL8 level increase was observed. Notably, the increase is minimal as compared to that recorded in healthy volunteers dosed with a competitive, orthosteric CXCR2 inhibitor [16], in keeping with the different mechanism of action (i.e., allosteric versus orthosteric). No statistically significant changes were recorded for the other cytokines measured (i.e., IL-1β, IL-6, TNF-α, GM-CSF, VEGF, b-FGF) (Additional file 1: Supplemental Materials and data not shown).

### Polymorphonuclear neutrophil biology

Since CXCR1/2 are expressed on the surface of peripheral blood neutrophils, we determined whether reparixin affected neutrophil biology.

Expression of adhesion molecules on resting PMNs and PMNs activated with exogenous CXCL8 (Additional file 1: Supplemental Materials) were examined at baseline and on day 21 of the study. The percentage of CD18^+^ PMNs following in vitro stimulation with CXCL8 was significantly (*p* < 0.05 by non-parametric test but actual power approximately 60%) decreased after 21 days of reparixin treatment (Additional file 1: Figure S5), consistent with inhibition of CXCR2 [22–24]. Moreover, the production of inflammatory cytokines (CXCL8, IL-6, TNF-α, and IL-1β) by monocytes and PMNs without or with activation by LPS and/or CXCL8 was evaluated by flow cytometry. No statistically significant difference was found in these cytokines (data not shown).

## Discussion

This is the first report of a window-of-opportunity clinical trial of a CSC-targeting agent. Reparixin monotherapy appeared to be safe and well-tolerated, similar to what was observed in metastatic HER-2-negative breast cancer patients where reparixin was administered in combination with weekly paclitaxel. PK data were also consistent with those obtained in the metastatic setting [11], with rapid absorption, high bioavailability, and short half-life following oral administration. Different than other CXCR2 inhibitors [16, 17], reparixin administration led to neither a decrease in ANC nor an increase in CXCL8 serum levels. The reasons for such difference may be related, at least in part, to inhibition of CXCR1 in addition to CXCR2, and the allosteric mechanism of receptor inhibition, which does not hinder ligand internalization.

Serial biopsies were obtained from all patients for detecting CSC and pathway markers in primary tumor tissue.

First, a decrease in CXCR1^+^ cells (all viable cells or tumor cells) was observed in the majority of patients, suggesting inhibition of recruitment of CXCR1^+^ cells from the bloodstream into the tumor bed. Achievement of pharmacologically active concentrations of reparixin following oral administration is also suggested by the decrease of the CD18 expression on PMN after 21 days of treatment.

Second, a reduction in ALDH^+^ and/or CD24^−^/CD44^+^ CSC populations was recorded in a proportion of patients, more often in the CD24^−^/CD44^+^ CSC population. However, the possibility of effectively enumerating each CSC population may have been hindered by BC intratumoral heterogeneity coupled with very low numbers of cells and, in addition, by two CSC-related factors: (i) the observation that ALDH1^+^ CSC reside in the center while CD24^−^/CD44^+^ CSC are found at the edge of a primary breast tumor, raising the issue of sample bias, and (ii) the ability of CSC to transition from one phenotype (i.e., ALDH1^+^ or CD24^−^/CD44^+^) to the other [14]. In breast cancer, CD24^−^/CD44^+^ CSCs were evaluated by flow cytometry before and after treatment only in a neoadjuvant trial where, similar to our study, a proportion of patients showed no detectable CD24^−^/CD44^+^ at baseline [25].

The additional methods used to measure the reduction in CSC following reparixin administration provided little information. IF was faced with very low numbers of cells, while RT-PCR results showed little concordance with FC data. Regarding ALDH+ CSC, any discrepancies observed between RT-PCR and FC data about ALDH may be explained, at least in part, by several, non-mutually exclusive factors: (i) the laboratories performing the assays each received one core biopsy, i.e., they did not share the same core biopsy; (ii) while ALDEFLUOR assay measures the enzymatic activity of ALDH, RT-PCR provides a semi-quantitative measurement of ALDH1 protein expression; (iii) RT-PCR, unlike flow cytometry, is performed on the bulk cell population from a core biopsy with no possibility for exclusion of irrelevant cell population which might dilute the signal for a given mRNA (i.e., no laser capture microdissection); and (iv) evaluation of RT-PCR results is performed by comparison with the housekeeping gene GAPDH rather than ALDH1 in a healthy tissue core biopsy.

It has been described that inhibition of CXCR1 on CSC by reparixin prevents FAK phosphorylation and downstream signaling including AKT phosphorylation [4]. However, possibly due to the very low numbers of CSC in these early-stage breast tumors, and the absence of chemotherapy which may have heightened CXCL8- and FASL release, it was not possible to demonstrate a decrease in P-FAK and P-AKT since the baseline value assessed by IHC was 0 in most patients.

The pleiotropic effects on CXCL8 in cancer prompted investigations also on markers of angiogenesis and autophagy. As concerns angiogenesis, CD31 staining was negative in the majority of patients at both baseline and day 21. Autophagy is a common phenomenon observed in CSCs in tumor microenvironment and has been associated with CSC stemness, drug resistance, and invasiveness. Autophagy, therefore, is commonly monitored by researchers to determine cancer therapeutic outcomes [26–28]. The autophagy marker P62 was reduced at day 21 as compared with baseline in the majority of evaluable patients, suggesting induction of autophagy.

## Conclusions

Overall, this trial demonstrated that oral reparixin 1000 mg t.i.d. for 21 consecutive days has a good safety profile and pharmacologically active concentration can be achieved in the bloodstream. Furthermore, the possibility that reparixin reduces CSC and induces autophagy is suggested. However, the clinical relevance of a ≥ 20% reduction in CSC following a single 21-day course of reparixin was beyond the scope of this study and remains currently unknown.

In general, the very low numbers and plasticity of CSC pose a challenge to the window-of-opportunity study design [29], and our findings suggest that clinical endpoints should be applied from the outset in developing CSC-targeting agents. Following these results and the phase Ib study in metastatic HER-2-negative breast cancer [11], reparixin is being investigated in combination with weekly paclitaxel in a randomized, double-blind, placebo-controlled phase 2 study in frontline metastatic TNBC (NCT02370238).

## Supplementary information

**Additional file 1.** Provides supplemental methods and materials and related figures for core biopsy analysis, as referenced in this manuscript.

## Data Availability

All data presented can be found in the Clinical Study Report available at Dompé farmaceutici S.p.A., Milan, Italy.

## Abbreviations

AKT: Serine-threonine protein kinase (also known as protein kinase B (PKB))
ALDH: Aldehyde dehydrogenase
ALDH1: Aldehyde dehydrogenase 1 isoform
ANC: Absolute neutrophil count
AUC_last_: Area under the concentration-time curve calculated by the linear trapezoidal rule-time 0 to last sample with a quantifiable concentration *C*_last_ at time *T*_last_
BC: Breast cancer
b-FGF: Basic fibroblast growth factor
cDNA: Circulating deoxyribonucleic acid
*C*_max_: Maximum plasma concentration
CSC: Cancer stem cell
CXCL8: Interleukin 8
CXCR1: Chemokine receptor 1
CXCR1: Chemokine receptor 2
DAPI: 4′, 6-Diamidino-2-phenylindole
EMT: Epithelial-mesenchymal transition
EpCAM: Epithelial cell adhesion molecule
ER: Estrogen receptor
FADD: FAS-associated via death domain
FAK: Focal adhesion kinase
FASL: Fas ligand
FC: Flow cytometry
FOXO3A: Forkhead box O3
GAPDH: Glyceraldehyde 3-phosphate dehydrogenase
GMCSF: Granulocyte macrophage colony-stimulating factor
HBSS: Hank’s Balanced Salt Solution
HER-2: Human epidermal growth factor 2
HMH: Houston Methodist Hospital
IF: Immunofluorescence
IHC: Immunohistochemistry
IL-8: Interleukin 8
LPS: Lipopolysaccharide
MDACC: MD Anderson Cancer Center
MFI: Mean fluorescence of intensity
PBS: Phosphate-buffered saline
PgR: Progesterone receptor
PK: Pharmacokinetic
PMN: Polymorphonuclear neutrophil
PTEN: Phosphatase and tensin homolog
qRT-PCR: Quantitative real-time polymerase chain reaction
RBC: Red blood cells
RCF: Relative centrifugal force
RNA: Ribonucleic acid
RT-PCR: Real-time polymerase chain reaction
t.i.d.: Ter in die (three times daily)
T_1/2_: Terminal half-life
TEAE: Treatment-emergent adverse event
*t*_max_: Time to maximum plasma concentration
TNBC: Triple-negative breast cancer
TNF: Tumor necrosis factor
VEGF: Vascular endothelial growth factor
xg: Gravity at the Earth’s surface (unit of centrifugal force)
