# Correction to: A window-of-opportunity trial of the CXCR1/2 inhibitor reparixin in operable HER-2-negative breast cancer

**DOI:** 10.1186/s13058-020-01294-7

**Published:** 2020-05-20

**Authors:** Lori J. Goldstein, Raymond P. Perez, Denise Yardley, Linda K. Han, James M. Reuben, Hui Gao, Susan McCanna, Beth Butler, Pier Adelchi Ruffini, Yi Liu, Roberto R. Rosato, Jenny C. Chang

**Affiliations:** 1 Department of Medical Oncology, The Hospital of Fox Chase Cancer Center, Philadelphia, PA USA; 2 University of Kansas Medical Research Center, Fairway, KS USA; 3 Current address: Early Oncology Development, Bristol-Myers Squibb, 3401 Princeton Pike, Lawrenceville, NJ 08648 USA; 4 Tennessee Oncology, Nashville, TN USA; 5 Indiana University Simon Cancer Center, Indianapolis, IN USA; 6 Current address: Parkview Cancer Institute, 11141 Parkview Plaza, Suite 305A, Fort Wayne, IN 46845 USA; 7 Department of Hematopathology–Research, MD Anderson Cancer Center, Houston, TX USA; 8 Research and Development, Dompé farmaceutici S.p.A., 20122 Milan, Italy; 9 The Methodist Hospital Research Institute, 6445 Main Street, Houston, TX 77030 USA; 10 Sarah Cannon Research Institute, 250 25th Avenue North Suite 200, Nashville, TN 37203 USA

**Correction to: Breast Cancer Res (2020) 22:4**

**https://doi.org/10.1186/s13058-019-1243-8**

After publication of the original article [1], the authors identified two errors in the Author details:

1. Sarah Cannon Research Institute, Nashville, TN, should be added to Dr. Yardley’s affiliation in addition to Tennessee Oncology.
2. Dr. Yardley has provided services to multiple companies on behalf of Sarah Cannon Research Institute.
