# Supplementary material for: A window-of-opportunity trial of the CXCR1/2 inhibitor reparixin in operable HER-2-negative breast cancer
Source: Breast Cancer Res. 2020 Jan 10;22:4. doi: 10.1186/s13058-019-1243-8 (PMC6954543; doi:10.1186/s13058-019-1243-8)
Supplement: Supplementary file 1 — Additional file 1. Provides supplemental methods and materials and related figures for core biopsy analysis, as referenced in this manuscript. [file 13058_2019_1243_MOESM1_ESM.docx]

**Supplemental Materials**

*Cell suspensions from biopsies*

To obtain single cell suspension, samples were weighed and observed for appearance, then placed in digestion media and kept at room temperature. The specimens were then rocked for 1 hour at 185 rpm (1.5 hours for surgical specimens), then filtered through 70 µm filter. HBSS+ was added to rinse any residual cells from filter, and the specimens were centrifuged 350xg for 5 minutes at 4ºC. For RBC lysis, cells were resuspended in sterile cell culture grade water, and after 20 seconds, 10XPBS was added. The specimens were then centrifuged at 350xg for 5 minutes at 4ºC. Supernatant was removed. The rest of the specimen was then filtered with 40 µm sterile cell strainer. Cell concentration was measured using Invitrogen Countess.

*Polymorphonuclear Neutrophil (PMN) Biology*

The expression of adhesion molecules (CD11b, CD18, and CD66b) on PMNs was investigated. Typically, 100 µL of whole blood were incubated with 10nM CXCL8 for 10 minutes at room temperature to upregulate the expression of adhesion molecules. Untreated whole blood samples were included as control. We measured the percentage of PMNs that expressed CD11b, CD18, and CD66b as well as the Mean Fluorescence of Intensity (MFI) of each adhesion molecule as a measure of expression density by flow cytometry.

Intracellular cytokine production by peripheral blood monocytes and PMNs were assessed using a modification of previously published procedure [1]. Briefly, monocytes and PMNs in whole blood were activated with CXCL8 (10nM), LPS (10µg/µL), and LPS+CXCL8 for 2 hours at 37 °C before the addition of Brefeldin A (BFA) for another 3 hours to block the transportation of the intracellular cytokines from the Golgi to the cell surface. Thereafter, red blood cells were lysed with 1×FACSTM Lysing Solution (BD Biosciences) and the membrane of activated cells permeabilized with 1×FACSTM Perm 2 solution (BD Biosciences). Next, the activated monocytes and PMNs were stained with fluorochrome-conjugated specific antibodies to IL-1β, TNF-α, CXCL8, and IL-6 before the samples were analyzed by flow cytometry. The percentages of monocytes and PMNs that produced IL-1β, TNF-α, CXCL8, and IL-6 were recorded.

*Cytokine levels*

The concentrations of interleukin (IL)-1β, IL-6, CXCL8, tumor necrosis factor (TNF)-α, granulocyte macrophage colony stimulating factor (GM-CSF), vascular endothelial growth factor (VEGF), and basic fibroblast growth factor (b-FGF) were measured in sera obtained from patients on days 1 and 21 of the study.

In brief, serum cytokines levels were measured using a method, as previously published [2]. Serum samples were processed and aliquoted within 24 hours for archiving at -80 ˚C.  Samples were analyzed in batch using Milliplex bead kits (EMD Millipore Corporation, Billerica, MA) according to the manufacturer’s protocol with overnight incubations and an additional point added at the low end of the standard curve, and analyzed using a Luminex LX100 (Luminex Corporation, Austin, TX). BioPlex control and analysis software Version 5.0, (BioRad, Hercules, CA) was used.

1. Gao H, Lee BN, Talpaz M, Donato NJ, Cortes JE, Kantarjian HM, Reuben JM: Imatinib mesylate suppresses cytokine synthesis by activated CD4 T cells of patients with chronic myelogenous leukemia. Leukemia 2005, 19(11):1905-1911.

2. Wang XS, Shi Q, Williams LA, Shah ND, Mendoza TR, Cohen EN, Reuben JM, Cleeland CS, Orlowski RZ: Longitudinal analysis of patient-reported symptoms post-autologous stem cell transplant and their relationship to inflammation in patients with multiple myeloma. Leukemia & lymphoma 2015, 56(5):1335-1341.

**SUPPLEMENTAL FIGURE LEGENDS**

**Fig. S1**. Morning predose circulating neutrophil count vs time profiles of individual patients.

**Fig. S2**. FACS gating strategy to identify CD24^-^/CD44^+^ and ALDH^+^ CSC population, and CXCR1^+^ normal and tumor cells, from tumor core biopsies.

**Fig. S3**: Immunofluorescence of core tissue biopsies from a representative patient collected before (**A**) and at the end of the 21-day reparixin treatment (**B**). Two fields are shown for each time point. Images are presented as individual staining in the left panels; the center image represents the overlapping of all of the individual stainings. The arrows indicate the presence of the CD44^+^/CD24^-/low^, ALDH1^+^ cells

**Fig. S4.** Serum CXCL8 levels in individual patients on day 1 and day 21. Symbols represent individual patients.

**Fig. S5.** The percentage of CD18^+^ PMNs evaluated before and at the end of the 21-day reparixin treatment. (**A**) *in vitro* CXCL8 stimulation; (**B**) unstimulated. All results are presented as median with 95% CI. Symbols represent individual patients.

**Figure S1**

**Figure S2**


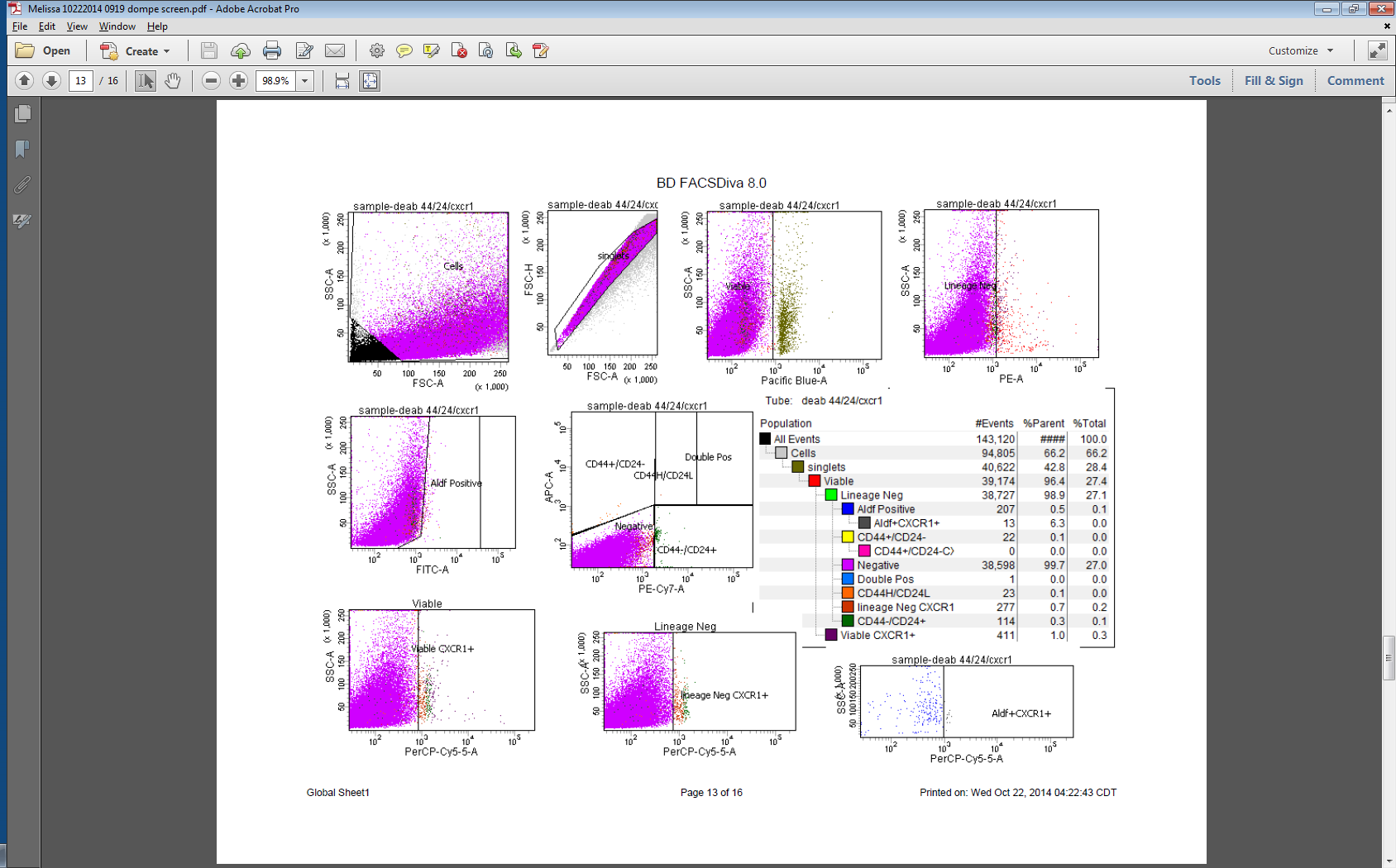


**Figure S3**.

**A**


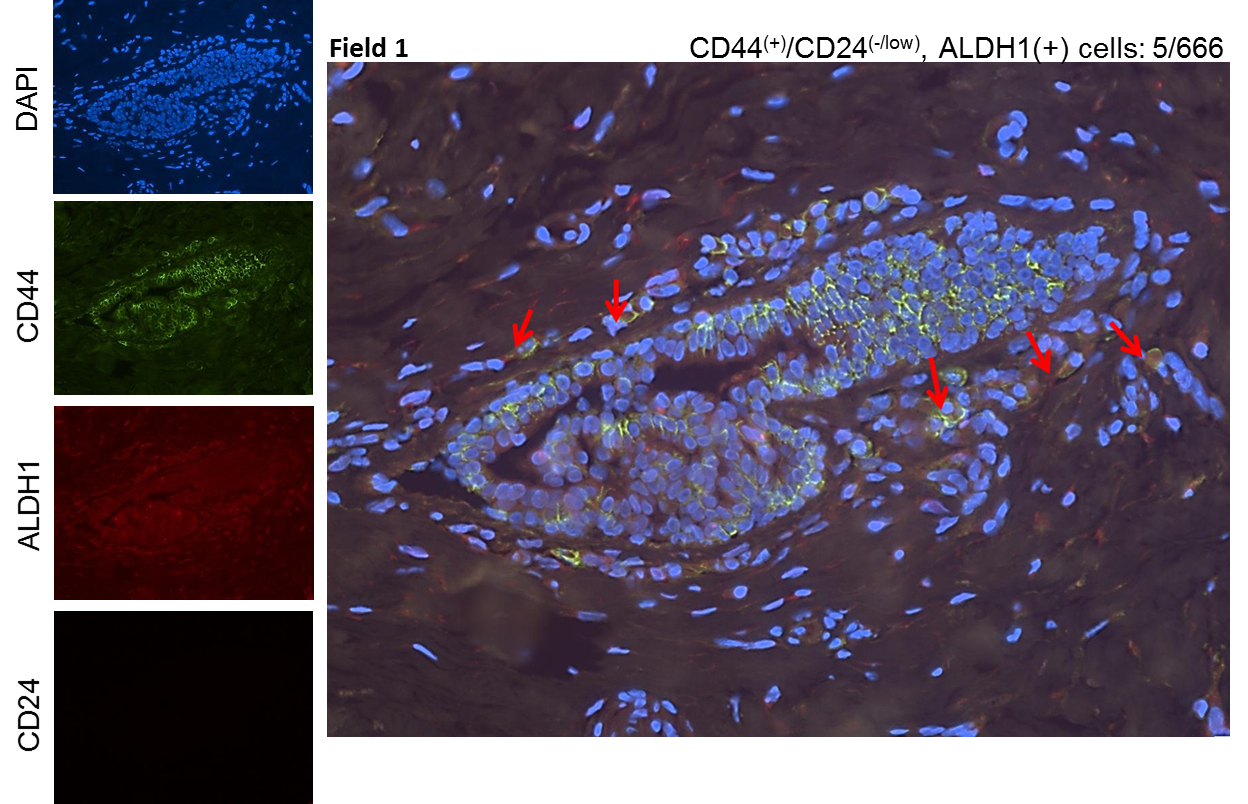

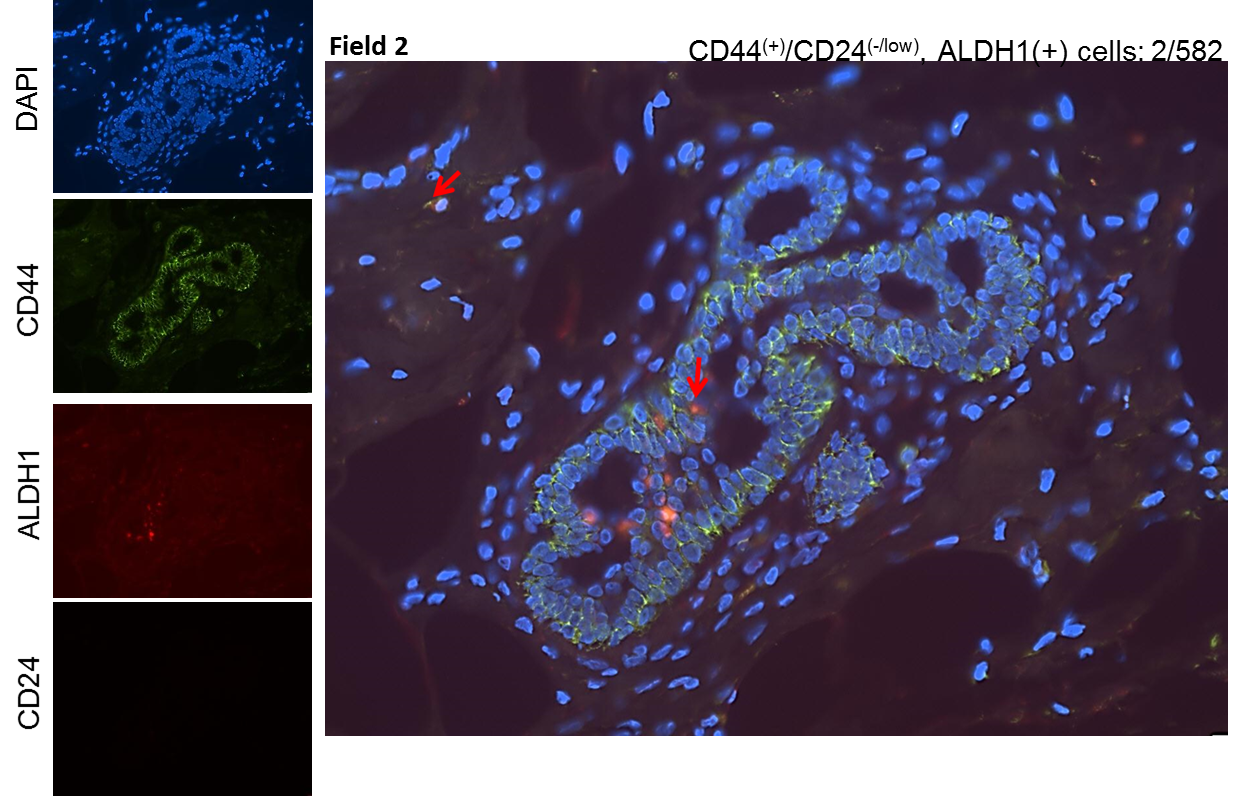


**B**


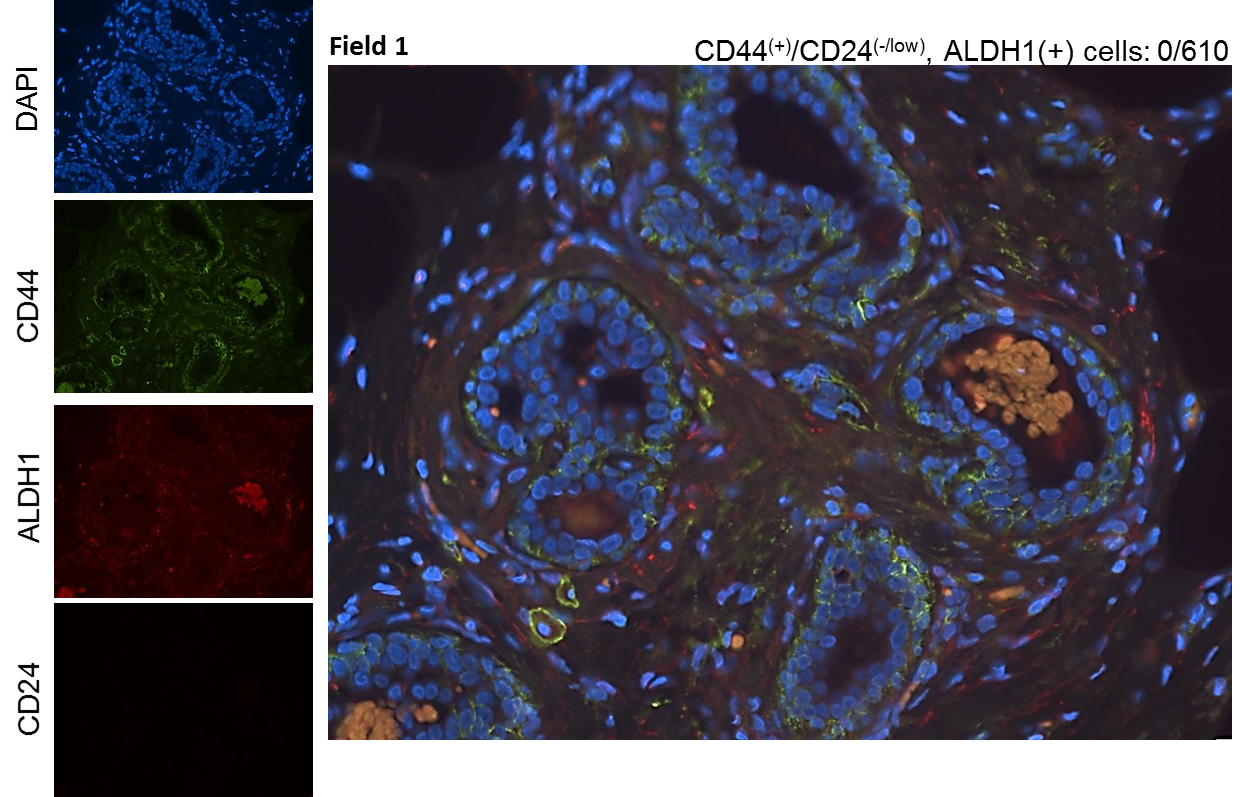

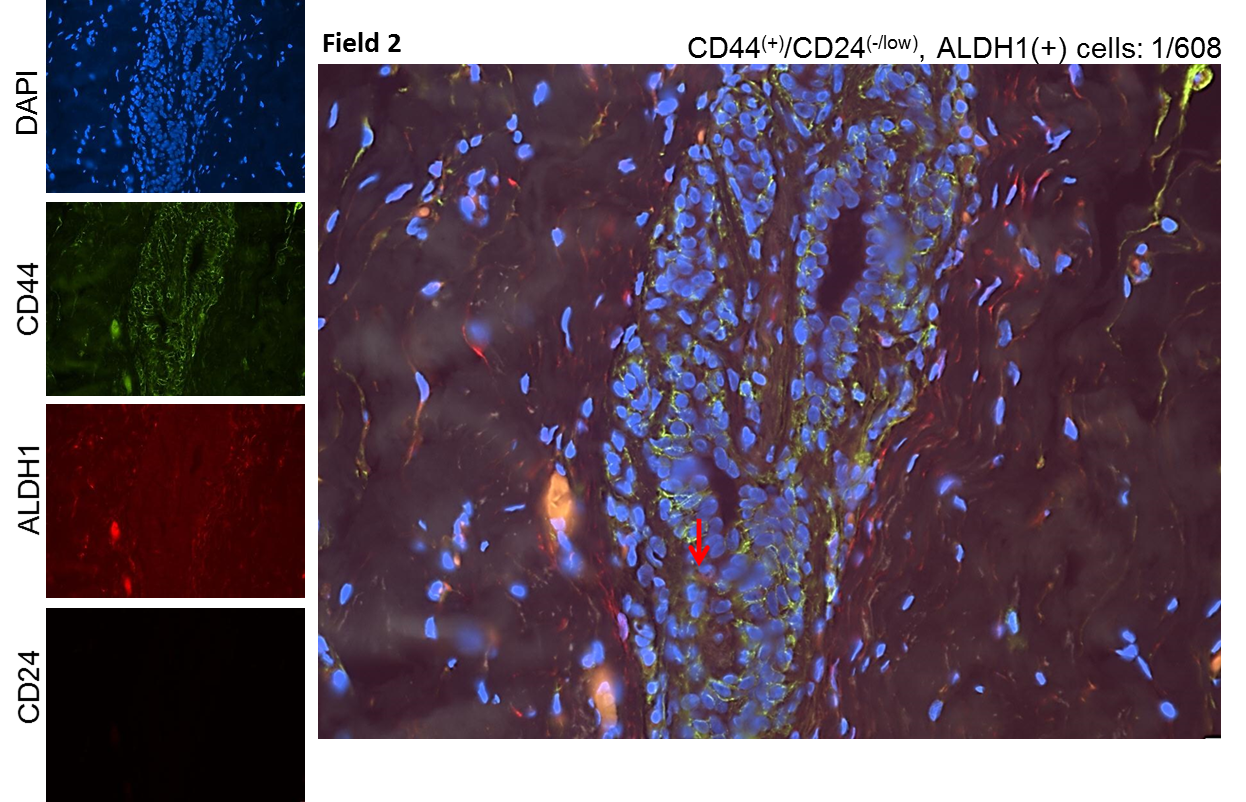


**Figure S4**


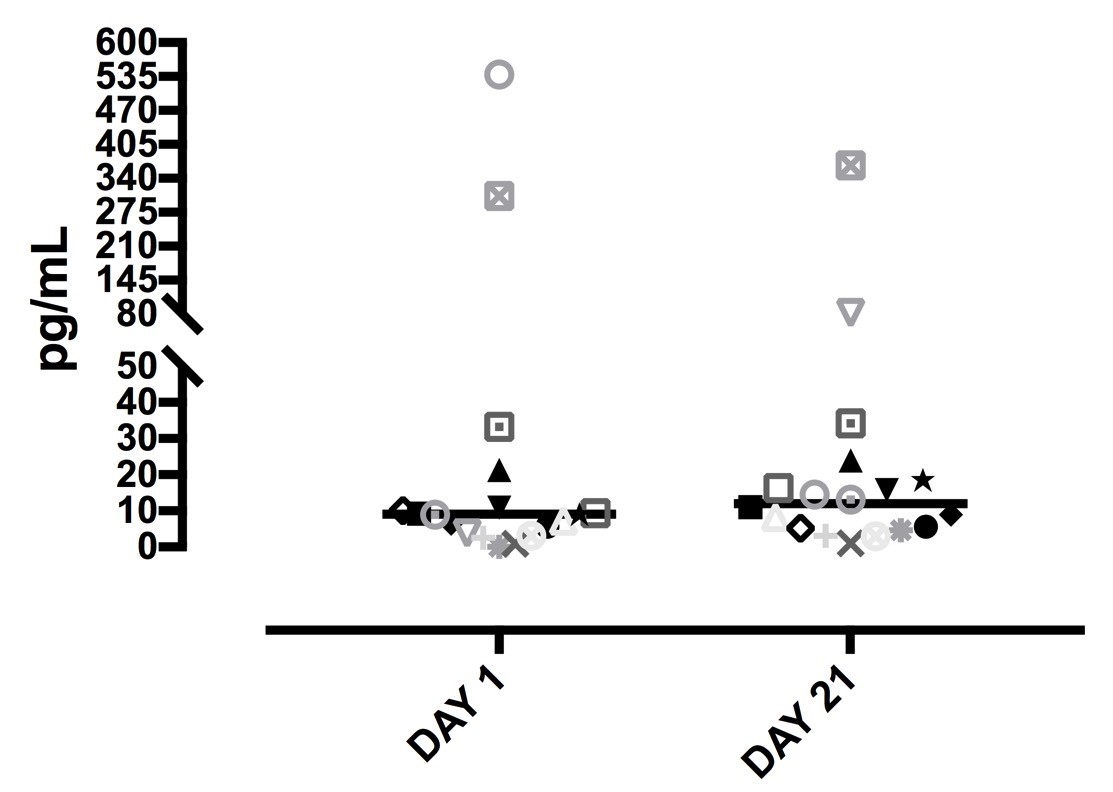


**Figure S5**

**
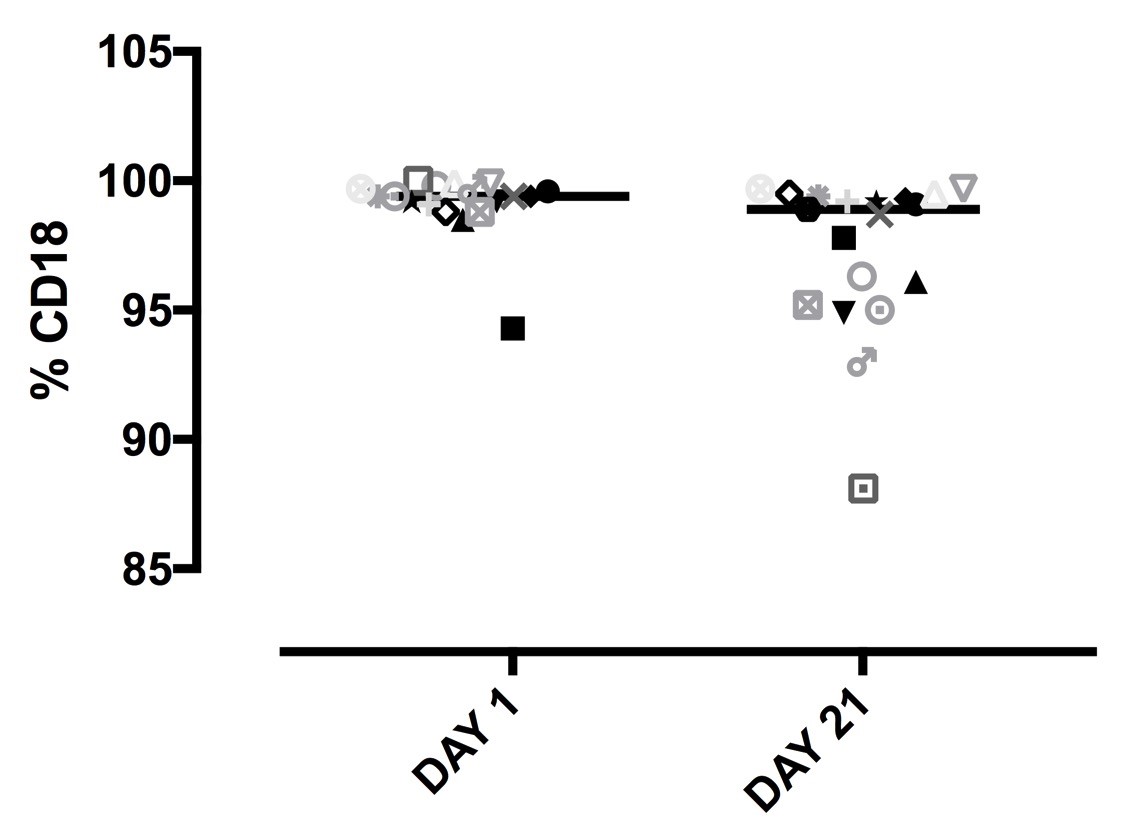
**
